# Experiments and Observations on the Pathological Alterations of Arteries by Ligature and Torsion

**Published:** 1846-07

**Authors:** 


### ART. VIII.

*Delle Alterazioni Patologiche delle Arterie per la legatura e la torsione, Esperienze ed Osservazioni di* LUIGI PORTA, Professore di Clinica Chirurgica nell' I. R. Universita di Pavia. Con tredici Tavole in Rame.—*Milano*, 1845.

*Experiments and Observations on the Pathological Alterations of Arteries by ligature and torsion.* By LUIGI PORTA, Professor of Clinical Surgery in the Royal University of Pavia. With thirteen copper-plates.—*Milan*, 1845. Folio, pp. 439.

BELIEVING that the alterations induced in the arterial system by the ligature had never been scientifically demonstrated, Dr. Porta took advantage of his situation in the University of Pavia to apply himself to the task, by experimenting on animals, and by gathering from his own practice and that of others, facts in human pathology illustrating the question. He commenced his experiments in 1835, and has continued them during nine years, making upwards of 600 upon 270 animals. Most of them have been made upon dogs, as these animals are more easily obtained, and offer a closer analogy in physical organization with man than others; but sheep, goats, horses, asses, oxen, and rabbits, have been also used. The greater number were instituted in the Clinical School with the assistance of students, and preparations in the museum are further proof of authenticity. The object of the author has been to solve the following problems:

1. What becomes of a ligature abandoned upon an artery; does the material of which it is formed influence the results of the operation, and, if so, what material is the most suitable?

2. What alteration does the artery undergo in the different methods of ligature and torsion, and which of the two methods merits the preference?

3. What change in the circle of the arteries succeeds obliteration of the principal trunks, and, consequently, what is the power of the arterial system in opening the channels necessary for the preservation of the limbs?

These inquiries are preceded by certain anatomical considerations upon the arteries. It appears that the arteries of the larger animals are much more tenacious than those of man, and those of dogs are one degree, and of goats and sheep two degrees, more fragile and weak. This, of course, varies with the age of the animal. We pass over a long description of the various coats of arteries, as we find nothing here peculiar to the author, until he comes to treat of the vascularity of the internal coat, when he argues that what is generally considered to be stellated injection of the capillaries which permeate this tunic proceeds from injection of the vessels of the middle coat being seen through the transparent internal one.

"Examining the larger arteries of the chest and neck in horses, dead of acute inflammation after ligature of the carotids, I have seen vessels morbidly injected on the external surface appear on the internal surface of the inner coat. This is more easily observed in the principal arteries of large animals, on account of the greater caliber of their *vasa vasorum*. In all these cases, however, if the artery be split, and the last fine layer of the internal tunic be removed by fine forceps in the situation of the injection, this layer is found to be without vessels." (p. 12.)

"Still vessels that do not exist, or that cannot be demonstrated in the normal texture, appear and may be shown after inflammation. I have already said that in dissecting large arteries, inflamed by the action of the ligature, we not unfrequently discern around them a network of small vessels, which pass from the cellular sheath, permeate the strata of the middle coat and appear on the external surface of the inner coat. Then if this coat be thickened, or if coagula and false membranes occupy the channel of the truncated artery, vessels are sometimes observed passing from the seat of the ligature, or traversing the walls of the divided vessel, and by a plastic power regenerating themselves in the bosom of the new formations. At times, also, within the clot of blood which plugs the artery, as I shall show hereafter, conspicuous vessels are prolonged which communicate with the external vessels. This phenomenon is much more rare and more difficult to demonstrate than is commonly supposed; but it offers a perfect analogy to what is observed in the nerves and mucous membranes, the ultimate layer of which never presents vessels in its texture, after injection of the finest coloured liquids; while the products of inflammation formed on the surface, lymph, cellular tissue, or false membrane, present vessels in large quantity, which spring from beneath, and are easily seen and injected." (p. 13.)

I. After these preliminary observations, the author passes on to describe his experiments made to determine the effects of ligature, and the influence which a diversity in its material exercises upon the artery to which it is applied. In his experiments upon the material of ligatures he has wisely confined himself to such as are most applicable in practice—catgut, silk, prepared and crude, flax and hempen threads, and hair—and as the results have shown no palpable difference between crude and prepared silk, or flax and hemp, the species of ligature are reduced to four—three of animal matter, catgut, silk, and hair; and one vegetable, flax or hemp. These substances were applied as simple threads upon the principal arteries of animals, by different processes, and the ligature left for various periods from a few days to three years. Among the number of experiments we shall state the result of the more important as briefly as possible.

Several experiments with catgut upon the carotids and femoral arteries of dogs and sheep are first related. They are of considerable interest, but we find it would be impossible, without the plates, to give any correct idea of the changes produced; and the conclusions of the author being apparently justly founded, probably this is all that most of our readers would require. These conclusions are thus stated:

"Among the substances applicable to the ligature of arteries, catgut is doubtless the most strong and homogeneous. A series of eighty experiments proves that even of small diameter it has sufficient strength to compress an artery, divide its proper coats, and maintain them in mutual contact until obliteration or division is perfect; but it differs from the other ligatures, as it does not always divide the internal wall at the moment when the knot is formed, but becoming lax by a process of softening, it sometimes allows the artery to reopen. This, however, does not interfere with the success of the operation, when the relaxation is gradual and the internal plug is formed in time.

"Still, from the experiments instituted, and partly related above, it appears that catgut a day, or even some hours, after its application, excites adhesive inflammation in the cellular sheath of the artery, and becomes covered by a stratum of plastic lymph, olivary in form, which surrounds and buries it. This lymph at first becomes modelled into a soft, gelatinous, adherent tissue, which gradually becomes atrophied, and generally leaves a simple cellular web; or a ligamentous substance more close and opaque, designed to isolate the ligature and the artery; or more rarely suppurative inflammation is set up in the seat of ligature, and a circumscribed abscess is formed when the ligature remains, until it is thrown off. The catgut relaxes, softens, dissolves, and becomes confounded with the surrounding cellular tissue; or the artery upon which it rested being consumed, it becomes dry and stiff, and lays bare in the layers of the cellular tissue; or becomes concealed in the peculiar tissue of the cord remaining between the truncated extremities of the vessel; or confined in a cyst, or coating of the plastic lymph which at first formed around it. The new cyst is at first simple and cellular, but afterwards it acquires consistence, and offers in its walls an external layer, strong aud coriaceous, and a species of epithelium, or internal rosy, soft, mucous layer, containing the ligature and some secreted matter. This cyst is generally small, resembling a grain of rice or millet, and when thus small it is ordinarily attached and closely adherent to the ligature, following all its irregularities, thus partly assisting in the process of solution. But sometimes, by exorbitant exhalation, it becomes enlarged to the size of a pigeon's egg, or more, then resembling a true encysted tumour. In its last changes, if not discharged by the fistula remaining after the operation, it withers and remains undisturbed among the tissues where it lies, or terminates by being with its cyst completely absorbed." (pp. 21, 22.)

The author took the necessary precautions to prevent the escape of the ligature, so that the fact of absorption was in many instances demonstrated. The plates contain several illustrations of each of these various effects of the catgut ligature. In thirty-three of eighty cases the catgut had completely disappeared, and in several others it was found so much softened that little doubt could be entertained of its progressive absorption.

More experiments were made with silk than with any other material, both in the manufactured and crude state, untwisting the threads of commerce and applying them not thicker than a human hair. The only difference in the results was, that manufactured silk left on an artery lost its artificial colour, while the unprepared maintained the white or yellowish colour natural to it.

When compared with catgut, silk ligatures produced much more irritation, and excited suppuration more frequently, although in many cases simple adhesive inflammation alone was produced. Like the catgut, they are first covered with plastic lymph, then connected with the cord remaining between the two extremities of the artery, or they lie bare between the layers of the common cellular tissue, or become encysted. If not thrown off, the silk may remain inert during life; yet in some cases the author says he has succeeded in demonstrating that after a long time it becomes softened, and may be absorbed. In one hundred and twenty experiments the ligature was discovered in one hundred and one cases, in nineteen (a sixth) it disappeared; in some of them it may have escaped observation, but as in most of those discovered the silk had undergone a gradual change, and great care was taken in the examination, the possibility of the absorption of silk cannot be denied.

No difference was found between slender ligatures of linen or hempen thread. They were not at all more irritating than silk. Of sixty cases, in three or four only did an abscess form, in all the others adhesion followed; and when they were applied merely as a loose knot, frequently, after the course of some months, they were found lying innocuous upon the uninjured and pervious artery. After an interval of two years, like catgut and silk, they are found either in a state of perfect integrity, or macerated, or completely removed. Fifty were found, ten had disappeared, and most of those found were in the stage of metamorphosis.

Forty experiments were made with black horse-hair, single and double, and the observations have been prolonged to three years. The only difference between the hair ligatures and the others is, that they preserve their physical properties unaltered, and still remain in the body innocuous. Of the forty ligatures thirty-eight were found. Of the two which disappeared, one was applied upon a sheep, and could not be found after seven months; whilst others of silk and thread, upon other arteries of the same animal, were found. The other was upon the carotid of a dog. It is much more probable that the ligatures escape unobserved than that they are absorbed, and thus regarded, they tend to confirm the correctness of the former observations. Human hair has been tied upon the arteries of rabbits, and, like horse-hair, has always been found unaltered.

During fifteen years the author has applied ligatures upon the principal arteries after amputation, wounds, or in cases of aneurism, and disease, in upwards of one hundred cases, and the comparative results in men and animals, with regard to the alterations in the ligature and its effects, show that suppuration is much more common in the human subject; that the ligatures, if left, almost always escape, sooner or later, either from the original wound or a secondary opening; but that in some cases of amputation, followed by union by first intention, when both ends of the ligature had been cut close off, they had remained in the limb and never appeared. Hennen, Veitch, and Lawrence have observed the same fact. Certain cases recorded by Porta and other authors tend to prove that when the ligature remains in the human body it undergoes the same series of changes as in the lower animals. The chapter is concluded by a table, which we give entire, as the results are practical and important:

LIGATURES APPLIED, 300.

1. *Quality.*

| | |
|---|---|
| Catgut | 80 |
| Silk | 120 |
| Thread | 60 |
| Hair | 40 |

2. *Methods.*

| | |
|---|---|
| Circular ligature | 140 |
| Loose ditto | 70 |
| Double ditto* | 45 |

3. *Animals—several experiments made on same animal.*

| | |
|---|---|
| Dogs | 65 |
| Sheep | 24 |
| Goats | 12 |
| Calves | 2 |
| Horses | 13 |
| Asses | 5 |
| Rabbits | 8 |

4. *Arteries (on 45 the double ligature was applied).*

| | |
|---|---|
| Carotids | 110 |
| Subclavians | 32 |
| Brachial | 14 |
| Iliac | 19 |
| Femoral | 80 |

5. *Time left.*

| | |
|---|---|
| 1 to 30 days | 44 |
| 1 to 3 months | 61 |
| 3 to 6 months | 48 |
| 6 to 9 months | 54 |
| 9 to 12 months | 46 |
| 1 to 2 years | 29 |
| 2 to 3 years | 18 |

6. *Result.*

| | | | | |
|---|---|---|---|---|
| Catgut | 80 | disappeared | 33 | |
| | | remained | | 47 |
| Silk | 120 | disappeared | 19 | |
| | | remained | | 101 |
| Thread | 60 | disappeared | 10 | |
| | | remained | | 50 |
| Hair | 40 | disappeared | 2 | |
| | | remained | | 38 |
| | | | 64 | 236 |

7. *Period of Disappearance.*

| | | Catgut. | | Silk. | | Thread. | | Hair. | |
|---|---|---|---|---|---|---|---|---|---|
| From 1 | to 30 days | 1 in | 10 | 0 in | 18 | 0 in | 11 | 0 in | 5 |
| 1 | 3 months | 4 | 18 | 1 | 21 | 2 | 16 | 0 | 6 |
| 3 | 6 | 6 | 16 | 2 | 17 | 1 | 10 | 0 | 5 |
| 6 | 9 | 8 | 13 | 4 | 25 | 1 | 9 | 1 | 7 |
| 9 | 12 | 6 | 11 | 5 | 19 | 2 | 8 | 0 | 8 |
| 1 | 2 years | 5 | 7 | 4 | 11 | 4 | 6 | 1 | 5 |
| 2 | 3 | 3 | 5 | 3 | 9 | 0 | 0 | 0 | 4 |
| | | 33 | 80 | 19 | 120 | 10 | 60 | 2 | 40 |

8. *Effects in the 236 ligatures which remained.*

| | |
|---|---|
| 1. Covered by lymphatics—cellular substance | 29 |
| 2. Attached to the cord between the ends of the artery | 60 |
| 3. In the common cellular tissue | 67 |
| 4. Encysted | 54 |
| 5. Forming abscess | 26 |

(pp. 40-1.)

* Two ligatures in each experiment.

This table is alone sufficient to show the care and industry of Dr. Porta; out we must pass without further comment to the succeeding chapter of his work, on the methods of ligature and torsion, and of the pathological conditions induced by them in arteries.

II. There are four methods of employing the ligature in the continuity of the trunk of an artery: 1, tying it round and leaving the ligature; 2, removing the ligature immediately after applying it; 3, interposing a foreign body beneath the ligature; 4, applying the ligatures and dividing the artery between them.

The common method of circular ligature is first treated, and a number of experiments are related, which were instituted for the purpose of determining the consecutive changes at different periods. Without the plates we could not convey any correct or clear idea of these changes in individual cases, and we must therefore be content, as before, with an analysis of the conclusions of the author, upon the result of one hundred and forty experiments:

"The circular ligature applied upon an artery determines the following mechanical alterations: division of the proper coats, strangulation of the cellular coat, and their approximation internally at each side of the ligature with the formation of a clot. In this condition the artery, in consequence of the operation, is subject to two kinds of inflammation, the adhesive and the suppurative. The experiments just related demonstrate the first kind, or obliteration by adhesion.

"The process of coalition of the tied artery has four periods: the first, external arteritis and organization of the *oliva cellulare;* [this implies a quantity of coagulable lymph exuded, covering the ligature, and taking the shape of an olive;] the second, closure of the vessel; the third, isolation of the closed extremities, and detachment of the ligature from the intermediate cord; the fourth, assimilation of all these parts.......

"The rapidity of the organization of lymph is extraordinary, beyond belief. At the close of the second to the third day the *oliva* is changed into a gelatinous tissue; and generally in less than a week the new cellular sheath is perfectly formed, and presents a most beautiful and beneficial provision of nature for the obliteration of the artery, and protection against the blood which threatens its future destruction." (pp. 53, 54.)

We pass over a minute account, more curious than important, of the other stages of the process, but extract a passage on the formation of the internal plug, or clot:

"The *trombo*, or internal coagulum, is among the first phenomena of the ligature of an artery, and arises, as it is known, from the stoppage of the blood between the ligature and the first lateral vessels. This purely physical phenomenon is general in tied arteries, but not so constant as to be observed in all cases. Of one hundred and forty experiments with the circular ligature the clot was not formed in twenty-nine, sometimes on neither side, and more frequently only on the side next the heart, or *vice versâ*, and this without neighbouring lateral vessels: several times the clot has not formed, and the artery has remained permanently empty, notwithstanding an interval of some *millimeters* from these vessels. When the clot forms it may take place in a few hours, or in the first day; or the blood may not be completely coagulated on the third or fourth day. The larger a vessel is, the nearer the heart, subordinate to the action of this organ, and rich in lateral branches, the longer is the clot in forming, it is weaker, or is altogether wanting. It is also erroneous to suppose that the clot is always formed in proportion to the capacity of the tied trunk, and to the distance of the first lateral branches; not unfrequently the clot is excessive, and passes beyond small and large lateral emissaries one or two *millimeters* in diameter ; while in other cases it remains short, or slender and thread-like, and only fills a part of the channel. When largest, it scarcely forms a plug, or completely fills the closed end of the artery, exactly uniting its walls......... Once formed the clot remains a long time unabsorbed, prolongs the process of assimilation, and it appears that it sometimes becomes permanent, traces being discovered several years after an operation; but when the artery goes through its last changes, the coagulum becomes white, hard, firm, almost calcareous, attenuated, forms part of the ligamentous walls of the vessel, and then entirely disappears." (pp. 56, 57.)

Travers and Jones believed that a division of the proper coats of the artery was necessary to the success of the operation, but our author states that it is not indispensable. In many of his experiments, particularly with catgut, he found that he had not divided these coats ; yet the channel of the artery was closed, and the result the same as in case of division. The obliteration then occurs in two modes : either by coalition and simultaneous assimilation of all three coats at the spot tied ; or by a slow and insensible contraction, which, after the obstruction of the clot, without destruction of the walls, renders the arterial tube impervious, and converts it in time into a ligamentous cord. But more frequently the ligature which does not at first mechanically divide the arterial tube, does so afterwards by the inflammatory process.

Besides obliteration by coalition, an artery may be closed also by suppuration. This is uncommon, however, in the lower animals, and is generally attributed to violence in the operation, motion, the introduction of foreign bodies, &c. Most commonly a little pus is formed in the centre of the lymph effused around the ligature, and this pus is reabsorbed. Complete suppuration without lymph is much more rare. When it occurs the tied ends of the artery lie in the middle of an abscess, separated from each other, with their coats soft and yellowish, and the opening of the canal closed only by the clot, which is the sole protection against hemorrhage for one or two weeks. As the suppurative process ceases, granulations spring up from the cellular coat and cellular tissue around the ends of the artery, and by their contraction the coats of the artery are brought together, and the opening is permanently closed. The artery, when thus cicatrized by the second intention, afterwards presents the same conditions as if it had united by primary coalition. A number of cases from the author's experience, and that of others, are given to show that the same changes occur in the human subject. But—

"The two kinds of inflammation above described have an inverse ratio of frequency in man and in brutes. It is very rare, and only from extraordinary circumstances, that a complete suppuration with total separation of the ends of the tied artery occurs in brutes; and it is rare that a perfect coalition is effected in our arteries. And as this is the most simple, ready, and prompt mode of closing vessels, we see how much more fortunate animals are than man in this respect.

"Operating in whatever manner, we can never expect in surgery the fortunate results from the ligature of arteries that are obtained in the lower animals; but, judging from analogy and from known data, I believe that when ligature of arteries shall be performed in man with greater delicacy than has been hitherto employed, selecting the most homogeneous material and the most simple method, respecting the cellular matrix of the vessel, cutting off the ends of the ligature, reuniting the wound, avoiding proximity to the aneurism, and enjoining the most perfect quiet to the patient, we shall obtain adhesion more frequently; and when, notwithstanding our care, suppuration comes on, it will be circumscribed, and not always prevent a plastic exudation which can close the mouths of the artery, and prevent those fearful accidents which, very rare in brutes, have so often put the life of man in danger." (p. 74.)

We now pass to a consideration of the other methods of tying arteries: 1, temporary ligature; 2, mediate ligature; and, 3, double ligature, with division of the vessel.

1. The method of *ablation* may be defined thus: the removal of a circular ligature immediately after its application upon an artery, and closure of the wound. In order to determine if this method was sufficient to excite inflammation of the coats of the artery, and thereby obliterate its canal, the author made a number of experiments, which he records. They were fifty in number, and made upon the principal arteries of various animals, dividing in every case the proper coats of the vessel with the ligature, and removing it at various periods from a minute to some hours. Division of the proper coats, and strangulation of the cellular, is produced, and the latter becomes inflamed for some lines in extent, and causes a lymphatic exudation, becoming restored to its normal state about the third or fourth week; very rarely the inflammation is greater, and the same coat is thickened, hardened, and contracted, and, remaining so, compresses the other coats, and diminishes the caliber of the vessel; suppuration is rarely produced.

Immediately after the removal of the ligature the tube is free to the current of blood, and is permanently pervious, uninfluenced by the division of the proper coats of the artery; while, on the other hand, these coats do not appear to be influenced in the changes they undergo by the sanguineous current incessantly passing along. It is only when the cellular coat is thickened, as before described, that the caliber of the vessel is sensibly diminished. Still, in some rare cases, a clot forms which obliterates the vessel, or arteritis follows, and external exudation of lymph produces the same result. Of the fifty cases, in six or seven a clot formed, and in five or six, lymph, or false membrane, which occupied totally or partially the canal of the artery.

Mr. Travers had preceded the author in these experiments. He left the ligature from two to twenty-four hours; and in eleven experiments upon the carotids of horses and asses he almost always obtained obstruction of the vessel by sanguineous or albuminous coagulum. His papers in the fourth and sixth volumes of the Medico-Chirurgical Transactions are well worth attentive perusal. Dr. Porta, in addition to the fifty trials above noticed, made twenty-four others, removing the ligatures at two, six, and twelve hours. In one or two cases obliteration followed, but in the majority it did not occur; the clot and coagulum were wanting, and the caliber of the vessel was not diminished. Travers, Copeland, Roberts, and Crampton have adopted this method in some cases of aneurism in the human subject. In one case a popliteal aneurism remained pervious after ligature for twenty-six hours, another after six hours; one of aneurism at the elbow was cured by ligature of the brachial removed after forty hours, and another after popliteal aneurism of the femoral removed after twenty-four hours.

Dr. Porta has also performed seventy experiments to determine the value of a loose or slack ligature. The kind of animal, the material of the ligature, and, above all, the degree of tightness of the ligature, have greatly influenced the results; but the sum of the whole may be said to be that either by clot, exudation of lymph, or slow contraction from thickening of the cellular coat, the artery was closed in half the cases; in the other half it remained pervious, without any, or with only very slight, alteration in its texture; while, in a few cases, a part of the walls became ulcerated or eroded, and gave rise to fatal hemorrhage. These experiments upon the loose ligature illustrate the effects of the ligature of reserve, which was at one time the fashion, as a supposed safeguard.

2. The *mediate ligature* has for its object the closure of the artery without injury to its coats. It is termed also flat ligature, ligature with a small cylinder, Scarpa's ligature. For the ligature a small riband is generally used, formed of several simple waxed threads, and as the intermediate body a small cylinder, or small roll of adhesive plaster.

To determine the value of this method the author made one hundred and twenty experiments—thirty-five with the permanent, and eighty-five with the temporary ligature. He has also collected ninety-six cases of mediate ligature performed upon the human subject, that both in its permanent and temporary application the results in man and the lower animals are similar. The following table will show these results in the eighty-five experiments upon the temporary mediate ligature:

| 1. *Animals.* | |
|---|---|
| Dogs | 44 |
| Sheep | 12 |
| Horses | 15 |
| Asses | 5 |
| Mules | 5 |
| Oxen | 4 |
| **2. *Arteries.*** | |
| Carotids | 40 |
| Subclavian | 20 |
| Brachial | 11 |
| Femoral | 14 |
| **3. *Period of Removal.*** | |
| 1 day | 9 |
| 2 days | 15 |
| 3 days | 43 |
| 4 days | 13 |
| 5 days | 5 |

| 4. *Condition of the Artery.* | | |
|---|---|---|
| Artery found uninjured | | 33 |
| a little eroded | | 21 |
| eroded and cicatrized | | 9 |
| divided into two portions | | 16 |
| reduced to a cord | | 6 |
| **5. *Condition of Canal.*** | | |
| Artery found pervious— | | |
| Carotids | 14 | 24 |
| Subclavian | 6 | |
| Brachial | 2 | |
| Femoral | 2 | |
| Artery closed by plug | | 34 |
| by lymph | | 11 |
| divided in two | | 16 |

The exact result of the mediate permanent ligature is not given; but the author states that, instead of being more secure than the common circular thread, suppuration is much more common, the artery is deprived of its cellular coat, and the internal clot is the only safeguard against hemorrhage.

3. The method of double ligature, with division of the artery between, was produced in forty-five cases—thirty-five with simple incision, and ten with removal of a portion of the artery of various lengths. The *mechanical* results are the same as in the common method, with the addition of ecchymosis, retraction of the divided extremities, and impulsory movement of the cardiac extremity. The first *vital* phenomenon after the operation is inflammation of the cellular coats, with copious exudation of lymph, which afterwards becomes modelled into a cellular cylinder, and restores the continuity of the vessel. This cylinder varies from a few lines to two inches in length, according to the degree of retraction after division of the artery. At first it is much more voluminous than the vessel, but afterwards it becomes smaller, firmer, and fibrous, and is reduced to the state of a thin ligamentous coat; or it is completely absorbed, leaving the ends of the artery permanently separated; or very rarely (it only occurred in two experiments) the closed extremities of the artery are united by a new cellular tube, in the midst of which the two ligatures are found.

In ten experiments made upon the carotids of dogs, with removal of a portion of artery between the two ligatures, the cylinder formed, as in simple division when the portion removed was very small, but when it exceeded two centimeters the cylinder was not found, and the extremities cicatrized separately without union between them.

In the forty-five experiments with the double ligature, hemorrhage and corrosion of the cardiac extremity occurred in three cases, which was never observed after the common ligature. A table of the results of six hundred ligatures in the human subject, which we shall presently relate, will be the best test of the practical value of the method. The conclusions of the author are as follows:

"The temporary and loose ligatures are simple methods, destined, from their inefficiency, to remain neglected. The permanent mediate ligature, formerly recommended from prejudice, should meet the lot which the same method in wounded arteries for a century has suffered. The temporary ligature, although at first sight tempting, after the above demonstrations, and the facts drawn from other authors, may be declared to be a difficult, complicated, and uncertain method. To this the double ligature adds violence, and a multiplicity of proceedings, frequent impossibility of execution, copious suppuration, and greater tendency to hemorrhage. Thus the circular ligature alone remains, to which good sense and practice have already accorded the preference, now approved and justified by science." (pp. 108-9.)

The tabular view of six hundred ligatures of the large arteries in man has been prepared with so much care, and affords so much information, that we give it entire:

1. *Operators.*

| | |
|---|---|
| Anglo-American | 275 |
| Italian | 131 |
| French | 108 |
| German | 64 |
| Russian | 22 |

2. *Sex.*

| | | |
|---|---|---|
| Male— | | |
| Adults | 545 | 551 |
| Children | 6 | |
| Female— | | |
| Adults | 48 | 49 |
| Child | 1 | |

3. *Indications.*

| | |
|---|---|
| Aneurisms | 418 |
| Aneurismal varices | 30 |
| Nævi | 34 |
| Various tumours | 19 |
| Wounds | 84 |
| Ulcers | 8 |
| Hemorrhage | 4 |
| Neuralgia | 1 |

4. *Arteries.*

| | |
|---|---|
| Aorta | 4 |
| Innominata | 8 |
| Carotids | 132 |
| Subclavian | 74 |
| Brachials | 68 |
| Common iliac | 11 |
| Internal iliac | 12 |
| External iliac | 96 |
| Common femoral | 16 |
| External femoral | 180 |

5. *Number of Arteries tied in the same Individual.*

| | |
|---|---|
| Two common carotids, right and left | 5 |
| Right carotid and subclavian (method of Brasdor) | 4 |
| Right and left external iliacs | 1 |
| Right and left external femoral | 7 |
| Right external iliac and left external femoral | 1 |
| Two common carotids, uncertain cases of Bünger and Cross | (2) |
| Two external femorals, uncertain cases of Hodgson | (2) |

6. *Methods.*

| | |
|---|---|
| Hunter's method (more than one ligature) | 23 |
| Brasdor's | 31 |
| Double ligature | 67 |
| Mediate ligature | 96 |
| Circular ligature | 356 |
| Unknown | 27 |

7. *Progress.*

| | |
|---|---|
| Regular, in cases | 381 |
| Anomalous, in | 219 |

8. *Accidents.*

A. Local Accidents.

| | |
|---|---|
| Hemorrhage from the tied vessel | 75 |
| Hemorrhage, suppuration, and gangrene from the tumour or wound | 40 |
| Return of the aneurism | 25 |
| Reproduction of aneurism in other parts | 11 |
| Gangrene of the limb | 49 |
| Palsy and atrophy of the limb | 14 |
| Phlegmon and local abscess | 13 |

B. General Accidents.

| | |
|---|---|
| Gastrico-bilious and typhoid fevers | 10 |
| Inflammation of— | |
| Head | 18 |
| Chest | 22 |
| Abdomen | 6 |
| Neuroses— | |
| Apoplexy | 2 |
| Tetanus | 3 |
| Convulsions | 1 |
| Syncope | 1 |
| Hemiplegia | 4 |
| Neuralgia | 7 |
| Cachexiæ— | |
| Hydrothorax | 1 |
| Tabes purulenta | 6 |
| Hemorrhage (Deaths, 3.) | 4 |
| Neuralgia (Deaths, 0.) | 1 |

9. *Result.*

| | |
|---|---|
| Cures | 433 |
| Deaths | 167 |

General mortality, 27 and a fraction per cent.

A. Mortality in Sex.

| | |
|---|---|
| In males | 551 |
| Deaths | 153 |
| In females | 49 |
| Deaths | 14 |

B. Mortality in indication.

| | |
|---|---|
| In aneurisms (Deaths, 117.) | 418 |
| Aneurismal varices (Deaths, 5.) | 30 |
| Nævi (Deaths, 3.) | 34 |
| Various tumours (Deaths, 8.) | 29 |
| Wounds (Deaths, 28.) | 84 |
| Ulcers (Deaths, 3.) | 8 |

C. Mortality from Artery.

| | |
|---|---|
| Aorta, cases (Deaths, 4.) | 4 |
| Innominata (Deaths, 8.) | 8 |
| Carotids (Deaths, 35.) | 132 |
| Subclavian (Deaths, 27.) | 74 |
| Brachial (Deaths, 10.) | 68 |
| Common iliac (Deaths, 5.) | 11 |
| Internal iliac (Deaths, 4.) | 12 |
| External iliac (Deaths, 22.) | 96 |
| Common femoral (Deaths, 8.) | 16 |
| External femoral (Deaths, 45.) | 180 |

D. Mortality in different Methods.

| | |
|---|---|
| Hunter's cases (Deaths, 10.) | 23 |
| Brasdor's (Deaths, 19.) | 31 |
| Double ligature (Deaths, 11.) | 67 |
| Mediate ligature (Deaths, 23.) | 96 |
| Circular ligature (Deaths, 102.) | 356 |

10. *Cause of Death.*

| | |
|---|---|
| Hemorrhage from the tied artery, in cases | 30 |
| Hemorrhage, suppuration, and gangrene from the tumour or wound | 17 |
| Bursting of internal aneurisms | 8 |
| Severe inflammation of the limb operated upon* | 30 |
| Progress of the disease indicating the ligature, tumours, wounds, ulcers, hemorrhage | 12 |
| Adynamia and vital exhaustion from severity of the operation | 13 |
| Fevers of different character, not evidently local in their origin | 6 |
| Internal inflammations of— | |
| Head | 12 |
| Chest | 20 |
| Abdomen | 6 |
| Neuroses— | |
| Apoplexy | 2 |
| Tetanus | 3 |
| Convulsions | 1 |
| Syncope | 1 |
| Cachexiæ— | |
| Hydrothorax | 1 |
| Tabes purulenta, from suppuration of the wound made in the operation | 6 |

* These thirty patients died from the direct effects of gangrene, and not from the amputation reequired by this occurrence.

We now pass to the article upon what the author calls the accidents of the ligature, meaning the unusual circumstances which may arise and disturb the course of the operation, or of the subsequent cure. The most important of these are arteritis and hemorrhage.

1. *Arteritis,* as ordinarily excited by the ligature, is moderate and defined in its extent; but it may be violent and diffuse, and cause great danger. In this case the three coats are found intimately united, as it were fused together; the cellular coat is thick and pulpy, rich in small vessels, and infiltrated with lymph or serosity. The proper coats are rarely swollen, but they become soft, tender, fragile, with very slight cohesion, and so easily torn that their separation is almost impossible. They maintain their colour, or sometimes put on a rosy tint, independent of vessels, which are only found in the false membrane on their surface. The internal surface of the vessel does not always preserve its natural polish, but becomes unequal and scabrous or undulated and flaky, from evident hypertrophy of the membrane and its abnormal productions. It is in these cases that blood-vessels have been discovered in it. The artery may retain its natural caliber, or become contracted, or may become dilated or enlarged in consequence of the simultaneous action of three causes—softening, hypertrophy of the walls, and impetus of the blood.

This arteritis may terminate in adhesion, more rarely in suppuration, and ulceration to varying extent. In the spots where the inflammation has been most intense, ulcers, on the walls, are seen of various size and shape, and a third or half of the tube may be implicated, and often departing from the point of ligature the ulceration destroys the artery to the extent of two, three, or four inches.

The best means of avoiding this accident is care in the operation, and quiet after it.

2. *Hemorrhage* after ligature arises from three principal sources—secondary opening in the tied vessel, an injured collateral branch, and from the aneurism, or wound, which has led to the operation.

In animals the danger from hemorrhage is trifling. In upwards of four hundred ligatures, followed by obliteration of the larger arteries, only three cases of secondary hemorrhage occurred, and five others in cases where the mediate ligature had been employed, with them being only two per cent.; while in man the table of six hundred cases shows that the proportion exceeds twelve per cent.

The condition of the artery is almost always the same—suppuration of the cellular sheath, perforation or division from corrosion of the proper coats, and partial or total deficiency of the internal clot.

The following table presents some important information. Of the six hundred cases, hemorrhage occurred in more than one hundred; but in upwards of forty the blood appeared from the wound, or aneurism, that had called for their operation, and the cases in which the artery gave way at the seat of the ligature amounted to seventy-five, or one eighth of the whole.

1. *Frequency.*

| | | |
|---|---|---|
| Ligatures | 600 | Hemorrhage in Cases, 75. |

2. *Arteries.*

| | | |
|---|---|---|
| Aorta | 4 | Cases, 0. |
| Innominata | 8 | Cases, 4. |
| Carotid | 132 | Cases, 9. |
| Subclavian | 73 | Cases, 9. |
| Brachial | 68 | Cases, 4. |

| | | |
|---|---|---|
| Common iliac | | 11 |
| | Cases, 3. | |
| Internal iliac | | 12 |
| | Cases, 2. | |
| External iliac | | 96 |
| | Cases, 6. | |
| Common femoral | | 16 |
| | Cases, 9. | |
| External femoral | | 180 |
| | Cases, 27. | |

3. *Methods.*

| | | |
|---|---|---|
| Hunter's | | 23 |
| | Cases, 5. | |
| Brasdor's | | 31 |
| | Cases, 7. | |
| Double ligature | | 67 |
| | Cases, 9. | |
| Mediate ligature | | 96 |
| | Cases, 15. | |
| Circular ligature | | 356 |
| | Cases, 39. | |

4. *Period.*

| | |
|---|---|
| 1 day | 1 |
| 2 days | 2 |
| 3 days | 3 |
| 4 days | 1 |
| 5 days | 4 |
| 6 days | 6 |
| 7 days | 1 |
| 8 days | 6 |
| 9 days | 2 |
| 10 days | 5 |
| 11 days | 1 |
| 12 days | 2 |
| 14 days | 3 |
| 15 days | 5 |
| 16 days | 3 |
| 17 days | 1 |
| 18 days | 2 |
| 20 days | 2 |
| 21 days | 1 |
| 22 days | 2 |
| 24 days | 3 |
| 26 days | 3 |
| 28 days | 1 |
| 30 days | 1 |
| 32 days | 1 |
| 33 days | 1 |
| 37 days | 2 |
| 42 days | 1 |
| 45 days | 2 |
| 55 days | 1 |
| 5 months | 1 |
| Time unknown | 3 |

5. *Result.*

| | | |
|---|---|---|
| Cases | | 75 |
| Cured | 31 | |
| Death from hemorrhage | 30 | |
| Death from other causes | 14 | |

Mortality of those in whom hemorrhage occurred, 55 per cent.

Mortality strictly caused by hemorrhage, 37½ per cent.

6. *Means of Cure.*

| | | |
|---|---|---|
| Cures | | 31 |
| With plug | 13 | |
| With torsion | 4 | |
| By second and third ligature of the artery | 9 | |
| By bandage and cold fomentation | 3 | |
| By amputation of the limb | 2 | |

III. The second section of the work is on the torsion of arteries. This is no modern invention; it was known to Galen and the Arabians, and was then practised almost as is done at the present day upon the smaller arteries, with a small hook or forceps. The instrument used by the author is a common pair of dissecting forceps, rather strong, with conical extremities, obtuse at the apex, the internal surfaces scored, and exactly joining along their whole length. On the large animals he employed large polypus forceps. No other instrument is necessary, and this is a principal recommendation of the method; as, if complicated instruments or proceedings were required, the operation not being an indispensable one, it could not be proposed in place of the simple, well known, and safe method of ligature.

The method is only applicable to wounded or divided arteries; and either simple torsion, or torsion after isolation of the vessel, may be employed. Simple torsion is adapted to small arteries of the fourth and fifth order; they are seized with small forceps, the instrument closed and turned in the fingers three, four, or six times, always in the same direction, and then the vessel is abandoned. In this way six, eight, or ten arteries on the surface of a large wound may be stopped in a very few moments. It may be necessary to repeat the torsion two or three times. The author frequently adopts this plan after amputations, upon the radial, ulnar, tibial and interosseal arteries. Previous isolation, and from seven to ten turns of the forceps, are necessary for the larger arteries. The errors to avoid in the operation are the seizing one wall of the vessel only, or not taking a sufficiently firm hold, seizing part of the surrounding textures with the artery, and twisting too little, so that the proper coats are not ruptured, or too much so that the cellular coat is also broken.

The author made sixty experiments on animals, and many observations in man, to determine the anatomical effects of torsion, and the subsequent alterations in the artery. They are closely analogous to those produced by the ligature, viz. circular laceration, and sometimes introflexion of the proper coats, strangulation of the cellular coat, and an internal clot, as mechanical effects; and adhesion or suppurative inflammation, as vital.

Several interesting cases, taken from the author's clinical practice, are given to illustrate the practical value of this method in the human subject. In the first, it was successfully employed in the brachial after amputation. No hemorrhage ensued, but the man died in three days from pneumonia; vessel closed by clot and adhesion. Same results in the second case, in a man who died four days after torsion of the tibial; and in the third, who died nine days after torsion of the brachial. In the fourth case, torsion was applied to the femoral after amputation of the thigh; the patient died of pulmonary abscess seventeen days afterwards; no hemorrhage occurred, and the artery was found perfectly closed. The same result occurred in a case in which torsion was applied to the popliteal after amputation; the patient died in ten days, also, from pulmonary abscess; and the popliteal was also closed in the sixth case, the patient dying of apoplexy fourteen days after the operation. In the seventh case, torsion was applied to the femoral after amputation; the patient died in twenty-one days of phthisis, and the artery was found exactly in the same condition as if a ligature had been applied. The eighth and ninth cases are instances of the successful application of torsion to the arteries of the leg and fore-arm after amputation. In the tenth case torsion was applied to the femoral; but as bleeding did not cease, a ligature was applied. A ligature was also necessary in the following case, in which torsion was used and repeated upon the femoral unsuccessfully. In the twelfth case the leg of a boy was amputated below the knee, the popliteal was divided above its bifurcation, and twisted six times upon itself. When the compression was removed the artery was seen to pulsate violently, turn twice round, and throw a jet of blood from its centre. It was again twisted three times, and the bleeding ceased. The wound cicatrized without any other accident. The other cases are three of successful application and termination of the femoral, and two to the popliteal after amputations. A case is also given in which a large varicocele had produced neuralgia and atrophy of the testis, and a cluster of the veins was removed of the length of two centimeters. Hemorrhage to a large amount followed from the mouths of five or six large veins in the cord. Each of them was seized and twisted five or six times, and a common bandage applied. No hemorrhage followed, and the cure was complete in a month.

The concluding paragraphs of this section are important, and we accordingly extract them.

"Since I introduced torsion into this school, I have almost exclusively practised it for the obliteration of wounded arteries. From the beginning I kept an exact register to the number of four hundred; then I neglected to record the daily cases in hospital and private practice, in which torsion was applied, except in cases of amputation. It was applied to the occipitals, temporals, maxillary, superior thyroids, some branches of the subclavian, the thoracic, external pudic, spermatic, &c., and to the digitals, so that there is no artery of the fourth or fifth order upon the surface of the human body that has not been repeatedly twisted, after accidental wounds or cutting operations, and the result of so much practice during nine years has been fortunate beyond every expectation. I well remember in some cases to have been obliged to repeat the twisting two or three times; but I do not remember that the ligature was ever necessary on account of torsion having failed. Thus I have ceased to employ the ligature for wounded arteries of moderate size, and now employ torsion only, as to the advantages of simplicity and celerity it unites others, as it may be done without assistance, and does not leave foreign bodies in the wound.

"With regard to the large arteries of the extremities, I have already applied torsion in sixty-five cases after amputations and disarticulations, principally performed in the Clinique, and some in private practice. Although the operation in these cases has not succeeded so fully and securely as in the preceding, yet in the majority it has answered. For the arteries of the fore-arm and leg, radial, ulnar, tibial, interosseal, torsion, either simple or after separation of the end of the artery, has constantly succeeded, except in two cases; in one of which the anterior tibial, and in the other, posterior tibial, gave way, and the ligature was required. On the brachial it has always succeeded; and of twenty-three operations on the femoral and popliteal it has failed in four cases, two already mentioned, the twisted portion, having given way, and ligature being necessary. I know that with some surgeons of my acquaintance laceration and hemorrhage have been more frequent, but that may depend upon error in method or management. Fricke has enjoyed very good fortune, having only one case of hemorrhage in thirteen torsions of the brachial, femoral, popliteal and tibial arteries. He, however, has no bounds to his enthusiasm, and prefers the new operation in all cases, and twists with success even ossified arteries, which are not susceptible of torsion. On the other hand, the cases of Delpech and Textor are inconclusive, and of no more weight against this operation than the first unfortunate trials of the Hunterian operation in France by Desault and Deschamps." (pp. 161-2.)

The author goes on to argue that ill success is more frequently to be attributed to incapacity in the operator than to the condition of the vessel. If not twisted enough, the current of blood will reopen it; if too much, the coats give way. He continues:

"The results of practical surgery corresponds with, and are even more favorable than, the results of zootomy. In my series of experiments upon the carotids, subclavians, and femorals of dogs, sheep, goats and horses, torsion in general succeeded; but in eleven cases out of sixty secondary hemorrhage occurred, requiring either a repetition of the torsion, or the ligature, and destroyed more than one animal. Experiments on brutes, then, do not give a secure criterion to argue the same results in man; because here torsion of the mouths of vessels upon the surface of the wound is a ready and simple proceeding, much more likely to be successful than torsion after exposing, isolating, and dividing the large arteries in animals." (p. 163.)

The conclusions are, that in wounds of arteries only, can torsion take the place of the ligature; and as it is perfectly safe and secure when practised on the smaller arteries, it ought to be performed when those arteries are divided. The question with regard to the larger arteries is not yet settled; but it would appear that although torsion answers in the majority of cases, still the ligature affords greater security against secondary hemorrhage.

IV. Having examined the changes of the ligature, and its effects upon the tied artery, the author commenced his investigations on the collateral circulation established after the obliteration of the principal arterial trunks. The collateral circulation, or anastomosis, he treats as direct and indirect; the first carried on by some vessels running directly between the extremities of the obstructed trunk; the second by muscular and subcutaneous branches. A number of very interesting plates exhibit the direct anastomosis at various periods, from a week to two years and a half. From them, and the narrative of the experiments and dissection, we learn that a direct communication between the extremities of a tied artery is carried on by means of branches arising from these extremities, and these branches are of two kinds, primitive and new. The first merely become enlarged; the second are generated in consequence of the application of the ligature.

"If an artery which has been operated upon be incorporated with another healthy one, in the first the roots of the *vasa vasorum* are found more capacious, numerous, and crowded than in the second. Clusters of vessels arise from the large lateral branches; and from the large clusters others smaller and smaller arise, which is not the case in healthy spots. The minute branches spring in preference from the small vessels, on account of the tenuity of their walls, the easy permeability of their pores, and the pressure of the blood that deviates from the tied trunk. Then some small vessel runs from the proper coat of the trunk, and runs isolated, or ramifies on the surface; this may be natural. But in some parts this vessel suddenly, at its origin, forms a shoot of small twigs, or presents a species of bud, or small bulb, from which small vessels spring in considerable number; this is doubtless preternatural. The effused lymph which forms a covering to the ligature is soon found very rich in vessels; and this abundance in a tissue newly formed around an artery could only be explained by new propagations from the turgid and dilated *vasa vasorum* of the cellular coat. After some time, upon the very apex of the closed extremity of the trunk, direct anastomotic arches are discovered; and to these anastomotic arches, in many cases, we cannot deny priority to the ligature, and their appearance in this place by simple enlargement. Those vessels that opened from the coats at some distance, by absorption of the obliterated extremities of the trunk, afterwards appear to run from the apex, being prolonged in a ratio with the retraction of the extremity. But the apex of this extremity is sometimes covered by tender buds: the closed end is cribriform and reopened by new mouths; and the vessels that these open into traverse from one extremity of the artery to the other, and are seen passing through the ring of the ligature that has strangulated and divided it. Lastly, within the cavity of the operated artery vessels are generated, to which the sanguineous plug serves as matrix, and these communicating with the external branches, enter into the system of direct anastomosis between the two extremities. I have not as yet discovered internal vessels, except in the carotids of dogs, some weeks or months after the ligature, because I have limited my observations to these arteries only; but I believe that the same thing may be verified in others. The phenomenon is more likely to occur in the carotids and external iliacs, because these arteries not dividing for a considerable distance evolve long coagula. However, I have seen new vessels formed in coagula only a few lines in length, which scarcely covered the blind extremity; and of the two ends of the carotid the inferior has much more frequently presented examples of internal vessels than the superior. At first I believed that the body become vascular in the cavity of the artery was always lymphatic or cellular; but reiterated observations have convinced me that it is generally the fibrinous clot, formed from the blood within the obliterated vessels, which is rendered vascular. Struck with the novelty of the fact, of which I had no previous idea, and not well assisted by injection, which succeeded imperfectly, I have maintained that the internal vessels advanced from the point, or were an appendix of the external anastomosis that arise from the arterial extremity at the base of the plug. [Instances are shown in plates.] By ulterior inquiries I have become certain that the internal vessels arise primitively within the coagulum or clot, and afterwards run to the apex of the closed artery, and become continuous with the external branches." (pp. 181-2.)

This direct anastomosis is much more easily demonstrated in the ruminating animals than in the carnivora, as in the former the collateral branches are less numerous, and, therefore, there are greater obstacles to a free re-establishment of the circulation. The human subject, from the number of lateral branches, resembles the carnivora; but still cases and plates are given, proving the occasional occurrence of direct communication.

Maunoir, Parry, Mayer, and Müller have preceded the author in this demonstration, but he deserves the credit of establishing the authenticity of the fact by the number of his observations, and of having made more exact preliminary studies of the normal state of the vessels, and of the pre-existing anastomoses; and by analysis of the whole process proved the new formation of vascular arches between the obliterated extremities of a tied artery. Many of the plates given in illustration are very beautiful.

The *indirect lateral* circulation is carried on by the anastomosis of secondary vessels pre-existing around the central obliterated trunk. These are deep or muscular, and superficial or subcutaneous. The author wished to determine exactly by what vessels the circulation was carried on in each region, and the changes they undergo. He demonstrates first the effect of the ligature of the femoral in different animals, as the anastomoses about the hip are so well defined, and the opposite uninjured limb offers so decided a contrast to the one operated upon; and then treats of the other principal arteries and their anastomoses. This division of the work occupies upwards of two hundred pages; but fortunately for us, as it consists in a great measure of details of the dissection of different animals, at various periods after ligature of the principal arteries, we need not follow the author very closely, as all that we wish is to put our readers in possession of the result of his labours in general terms. We shall not even descend to particulars, except with regard to the aorta, even in the human subject, as this part of the subject has been so well worked in this country, and the results are known to every surgical student.

The changes which the principal lateral branches undergo after ligature of their parent trunk consists in enlargement. It is clear that all the blood passed by the trunk is very soon, after the ligature, carried through the lateral branches. If a sensible quantity of blood were detained, diminished vitality of the limb would afford evidence of such detention. Now, such diminished vitality Dr. Porta has never observed in any of his very numerous experiments on animals. Yet, in opposition to this argument, the most careful examination of the dead body, during more than a week after the operation, does not show the least dilatation of the lateral branches; and when the dilatation does commence, it proceeds very slowly, and does not equal the caliber of the tied trunk for some months. This apparent contradiction is explained by the natural elasticity of arteries allowing an instantaneous dilatation during life, which cannot be detected after death. That such a vital dilatation does take place is proved by the following experiments :

"1. When the two carotids are laid bare in a living animal and one is tied, the other is very soon seen to become dilated and turgid, and to pulsate more strongly than before. In the same way the superior branches of the superscapular and mammary, when the subclavian is closed below by forceps or ligature, at once become dilated, and are seen to be enlarged, while after death no difference can be detected.

"2. When in dogs, sheep, or goats, a day or two after the ligature of the subclavian or superficial femoral, the superior branches of the operated trunk are laid bare, they are observed turgid and capacious, but this disappears, and cannot be shown after death.

"3. When in any of these animals, or in the horse, the two carotids are tied in the middle of the neck, and one is opened above the ligature, at first nothing occurs; but after fifteen or twenty seconds a jet of blood commences, which rapidly increases, and continues as if it were from the cardiac extremity. It is true that this experiment is not sufficient proof of the sudden enlargement of the lateral anastomosis, because the communications of the carotids within and around the cranium with the vertebrals are naturally large, short, and direct; but in the subclavian, external iliac, and superficial femoral, where the natural anastomoses are much more slender, the same experiment affords the same result, and after ligature the blood, from an incision of the artery below it, jets forth in quantity until the animal faints and dies.

"4. When the ventral aorta is tied in an animal, and the femorals are opened, hemorrhage does not take place at first, but after a few moments the blood issues from the wounded arteries in the same manner in which it comes from open veins; doubtless on account of the sudden dilatation which it has produced in the lateral branches, although these vessels, both in this and the preceding experiment, appear natural in the dead body." (pp. 217-18.)

"In animals with transparent arteries, as frogs, I have frequently seen under the microscope, when an arterial branch has been closed with forceps, that the adjacent branches suddenly enlarge, and the blood is urged on with greater velocity. From these facts we argue that in the first moments after the ligature of the principal arteries of the limbs, the blood is accelerated in the lateral vessels, and by this acceleration partly overcomes their temporary want of size." (p. 219.)

But it is difficult to suppose that an acceleration of the circulation and some mechanical distension of the later branches is sufficient to maintain the integrity of a part or organ, when the innominata, or the two carotids and a vertebral, or the common iliac are tied, or at any rate to supply the same quantity of blood as before the ligature. It is more reasonable to suppose that a limb, left motionless, may for a time be deprived, without injury, of a portion of the blood with which it was supplied in the healthy state in quantity sufficient for the performance of all its functions.

The more minute branches, or anastomotic arches, are shown to be enlarged from the first day, not only from the easier passage of injected matters, but also by the eye and the scalpel. The enlargement is thus first detected in the "median arches, then in the secondary ramifications, and, lastly, in the lateral trunks, which are inserted above and below the obliterated extremities of the artery." This may be explained by the greater thickness and resisting power of the larger branches.

When the circulation is established sufficiently to supply the necessities of the part, the vessels remain without further alteration ; and this occurs at a period varying from some months to a year after the ligature. In dissections made from twelve to thirty-six months afterwards, no further alteration was found in the anastomotic system.

We find some important observations in the section on the effects of ligature of the carotids. In upwards of one hundred cases of ligature of one or both carotids in dogs, sheep, and goats, disturbances of the nervo-muscular system, indicating a deficient supply of blood, were never observed. In thirty-two experiments upon horses, asses, mules, and calves, alarming phenomena occurred in five cases. In three horses the thread securing the second artery was scarcely tied, than extreme agitation, spasm of the jaws, great anxiety in the chest, tremors and convulsive motions of the limbs came on, and death soon followed. Two mules, whose carotids were tied at the same time, suffered from paralysis of the extremities, particularly the posterior ; they were obliged to be raised and kept from the ground by cords, on account of actual defect of muscular power. Their heads fell on the manger, they refused food and drink, and died in eighteen and twenty-four hours, while no recognizable alteration or injury to the nerves could be detected after death. The other nineteen cases were without any ill consequence. In calves, the operation was as safe as in dogs or sheep; the animal walks about and grazes immediately afterwards.

With regard to ligature of the vertebrals, Dr. Porta is of opinion that the weakness of the extremities, dyspnœa, and nervous symptoms, ascribed by Sir Astley Cooper to a defective supply of blood to the brain, are merely the results of apathy and moral depression, which follow every severe operation, and not attributable to cerebral anæmia. Sir Astley fancied that the phrenic nerves, especially, suffered deprivation of blood. The author argues that the anastomoses are so free that no want of blood is possible from simple ligature of the vertebrals. He has never seen the instant death, as described by Cooper, after compression of the two vertebrals ; but he has seen animals suffer greatly, and this was to be attributed to their delicacy, or the extreme difficulty and long duration of the operation ; and he believes that the vertebral arteries have no greater influence than the carotids upon the functions of the brain.

Eleven experiments were performed to determine the collateral circulation of the innominata after the ligature of the roots of the carotids and subclavian at each side ; but the operations were very disastrous, six deaths occurring from hemorrhage, thoracic inflammation, or violent convulsions. Of the five survivors, two had no bad symptoms ; in the other three, orthopnœa, from spasms or convulsions of the diaphragm, gradually disappeared in the space of a few days.

The common iliac was tied in sixteen dogs. Half died from acute inflammation around the spot, or from peritonitis ; one or two from adynamia ; but the mortality was increased by the animals biting away the dressings, and laying the parts bare.

The section on ligature of the aorta is an interesting one. Experiments were made on sixty animals—twenty-seven dogs, nine sheep, five goats, and nineteen rabbits; fifty-three died; that is, all the rabbits, sheep, and goats, and twenty dogs. Seven dogs recovered, but two afterwards died from causes independent of the ligature. The deaths from the "circular" ligature took place from a few hours to three days; from the loose ligature, not for a month. The causes of death in nine cases were abdominal inflammation, the direct result of the operation; the rabbits and several dogs died from the depressing effects of the operation upon the general system; the other animals from paraplegia, or general paralysis from a defective supply of blood to the parts below the ligature. Some experiments have shown that when an animal suffers greatly from the ligature, if he does not sink under the abdominal disease, he may be saved by removing the ligature, and allowing the artery to reopen. One of them is worthy of extraction. After the ligature in a dog, six months old—

"The pulsation in the iliacs and femorals instantly ceased; the limbs became sensibly colder, and in less than ten hours incapable of motion or supporting the body; so that, notwithstanding the efforts of the animal, he was compelled, against his will, to lie down. Twenty-four hours of obliteration of the aorta and paraplegia, with evident sinking of the general powers, having passed on, I laid bare the wound, divided the ligature, and removed it. Immediately afterwards the arteries resumed their natural pulsation, and the animal, rapidly recovering, was able to walk with security upon the four limbs. There were no abdominal symptoms, but the wound of the operation suppurated, and was not cicatrised until the end of three months. The animal was then killed, and the aorta was found entire and pervious, without rupture or corrosion of its coats, and girdled externally by a stratum of adherent cellular tissue, and internally by a small clot of lymph adherent to its walls, near the orifice of the left external iliac." (pp. 349-50.)

The mediate ligature was used in this experiment, a piece of cork being placed between the thread and the artery.

"Opening the femoral arteries suddenly after obliteration of the aorta demonstrates that the circulation below is maintained, and arrives through the small branches in the central trunk of the limb. Yet the hemorrhage from the wound in this trunk only expresses a fraction of the circulatory power, because, with the closure of the aorta, the femoral having ceased to be the centre of the circulatory system of the thigh, does not receive from the new centre of the small collateral anastomoses more than a fraction of blood, and a small impulse on account of its great caliber, and the remote and indirect action of the heart. It is certain that if the gastrotomy, indispensable in exposing the aorta, was not so frequently fatal, the ligature of this artery would more often succeed, the circulatory power of the lateral anastomoses being frequently sufficient to support life. But it is equally certain that in many animals this power is not sufficient to maintain life, and hence paraplegia, general paralysis, depression of the powers, and death." (p. 355.)

In the anastomoses the mammary, intercostals, colic, mesenteric, and first lumbar, have not been found to take the part their anatomical disposition would lead one to anticipate; the principal anastomotic chain being always formed by the four pairs of lumbar and posterior sacral nearest to the obliterated spot.

Although the deaths have exceeded three fourths of the number operated on, still the possibility of recovery in a certain proportion has been demonstrated. Dangerous and uncertain operations may be hazarded in desperate or fatal cases. New operations seldom succeed at first; the ill success of the few cases of ligature of the aorta in the human subject is, therefore, not a sufficient reason to reject the operation altogether; and, "perhaps in future, operating better in the left ileo-epigastric region, with simple displacement, and without tearing the peritoneum, in the same way that we tie the common and internal iliac, the dangers will be diminished, and the saving some patients will authorize us to admit in surgery a just indication for ligature of the aorta. I have composed the present article, with the express desire that this operation may hereafter prove useful, and at least afford in man the same results obtained in the lower animals." (p. 368.)

The last chapter of the work is on the "accidents of the lateral circulation." It may be deficient or excessive. Excess is not observed in the lower animals; but in sick men, plethora, inflammation, or a return of the aneurism, are often the result of a too active collateral circulation. Plethora and inflammation may appear in the part operated on, or elsewhere. In almost all limbs, some days after operation, heat, throbbing, pulsation of the superficial arteries, and local fever occur; but this is temporary and unimportant. But in the head, congestion and threatened encephalitis may, in a certain degree, be attributed to undue reaction of the anastomotic and capillary systems.

"Of seven cases of ligature of the carotid recorded, and four operated on by me, in five it was necessary to abstract blood repeatedly during the first days, to relieve the head from plethora; and this is singular, because in the one hundred and thirty-two cases of this operation, set forth in the table, mention of encephalitis is scarcely ever made. (p. 371.)

Dr. Porta then quotes cases by Abernethy, Langenbeck, Dohloff, and Wattman, in which evident marks of inflammation of the brain and its membranes were found after death, succeeding ligature of the carotid; and others by Landa, Wardrop, Magendie, and Barovero of Turin, in which the symptoms evinced a similar condition. But more frequently reaction in the head is owing to "reverberation" induced by obliteration of a distant artery. Many cases are quoted, in which cerebral inflammation has been found after death, or evinced by symptoms during life, after ligature of the subclavian, iliacs, and femorals. Ligature of the large external arteries has frequently occasioned inflammation of the viscera of the chest and abdomen, or of their investing membranes, probably proceeding in part from the "hydraulic disorder of the blood in the limb operated on," but more often from local inflammation and constitutional irritation. Still, of six hundred cases, in forty-six only has severe inflammation, either local or general, been set up.

A too free collateral circulation, and undue dilatation of the anastomotic branches, may induce a return of the aneurism. It will be seen by the table that this occurred in twenty-five of the four hundred and forty-eight cases in which ligature was applied for the cure of aneurism or aneurismal varix.

Paralysis, atrophy, or gangrene, may be the result of defective collateral circulation, and these results are much more frequent in man than in the lower animals. They frequently precede the operation, being effects of the compression exerted by the aneurismal tumour on the principal nerves of the limb; but either atrophy or palsy may occur separately, and as an immediate sequence of the ligature; or the atrophy may be one of the sequels of paralysis; or, again, may exist alone, without alteration of the nervous system, being a simple effect of defective nutrition, from insufficient supply of blood. Several instances of each of these results are recorded in the text.

It will be seen by the table that gangrene was observed in forty-nine of the six hundred cases; more than half of these were attacked from the second to the third day, others from the second to the third week. When circumscribed, it was limited to the fingers, hand, foot, or leg, and advanced slowly; when diffuse, its course was rapid and fatal. It is seldom the result of the ligature alone, but depends on local and general causes in combination—as ossification of arteries, severe wounds, great size or diffusion of the aneurism, hemorrhage, ligature of nerves, or bad operating, general debility, or the presence of disease in the system.

We here close our analysis of this work, and need say but little as to its merits, as our readers can form their own opinion from a perusal of the preceding pages. Still we cannot leave our author without giving expression to the high opinion of his industry and perseverance impressed upon us by his work. He laid down certain objects for investigation, and proceeded in his task in a philosophical manner, making experiments wherever they appeared necessary, and collating from the works and experience of others such facts and observations as were calculated to assist him in his researches. Although no results of any striking novelty have been thus obtained, yet much light has been thrown on the formation of direct anastomosis, and fair grounds have been afforded for regarding torsion more favorably in practice than hitherto. With regard, also, to the materials of ligatures, their form, application, permanent or temporary stay upon the artery, and their various ill effects, although the author's deductions from his experiments do not differ materially from generally received opinions in this country, still, by the number of his observations, he has afforded a far more solid basis than previously existed on which to found those opinions. It is not the least of his merits that he appears to have operated with so much care, so publicly, recorded his experiments with such good faith, and reasoned so soundly upon them, that many of the questions he has investigated may be regarded as finally determined, and, consequently, those who might have been disposed to follow in the same path may employ their time and energies in other departments of science. The plates are excellent specimens of art, and the letterpress is carefully got up. Altogether, the book is both a very beautiful and a very valuable one.

We trust this work may be received as a token of the regeneration of the Italian school of medicine, and that at length observation and experience will take the place of the fanciful hypotheses which have so long fascinated and misled our southern brethren. We cannot convey our opinion of the merits of this volume better or more briefly, than by saying, that its author is worthy the place he holds, as the successor of the great Scarpa.

